# Impaired Topographic Organization in Patients With Idiopathic Blepharospasm

**DOI:** 10.3389/fneur.2021.708634

**Published:** 2022-01-12

**Authors:** Yanbing Hou, Lingyu Zhang, Qianqian Wei, Ruwei Ou, Jing Yang, Qiyong Gong, Huifang Shang

**Affiliations:** ^1^Laboratory of Neurodegenerative Disorders, Department of Neurology, National Clinical Research Center for Geriatrics, West China Hospital, Sichuan University, Chengdu, China; ^2^Department of Radiology, Huaxi MR Research Center (HMRRC), West China Hospital, Sichuan University, Chengdu, China

**Keywords:** blepharospasm, resting-state functional MRI, graph theory, network, least absolute shrinkage and selection operator

## Abstract

**Background:** Idiopathic blepharospasm (BSP) is a common adult-onset focal dystonia. Neuroimaging technology can be used to visualize functional and microstructural changes of the whole brain.

**Method:** We used resting-state functional MRI (rs-fMRI) and graph theoretical analysis to explore the functional connectome in patients with BSP. Altogether 20 patients with BSP and 20 age- and gender-matched healthy controls (HCs) were included in the study. Measures of network topology were calculated, such as small-world parameters (clustering coefficient [*C*_p_], the shortest path length [*L*_p_]), network efficiency parameters (global efficiency [*E*_glob_], local efficiency [*E*_loc_]), and the nodal parameter (nodal efficiency [*E*_nod_]). In addition, the least absolute shrinkage and selection operator (LASSO) regression was adopted to determine the most critical imaging features, and the classification model using critical imaging features was constructed.

**Results:** Compared with HCs, the BSP group showed significantly decreased *E*_loc_. Imaging features of nodal centrality (*E*_nod_) were entered into the LASSO method, and the classification model was constructed with nine imaging nodes. The area under the curve (AUC) was 0.995 (95% CI: 0.973–1.000), and the sensitivity and specificity were 95% and 100%, respectively. Specifically, four imaging nodes within the sensorimotor network (SMN), cerebellum, and default mode network (DMN) held the prominent information. Compared with HCs, the BSP group showed significantly increased *E*_nod_ in the postcentral region within the SMN, decreased *E*_nod_ in the precentral region within the SMN, increased *E*_nod_ in the medial cerebellum, and increased *E*_nod_ in the precuneus within the DMN.

**Conclusion:** The network model in BSP showed reduced local connectivity. Baseline connectomic measures derived from rs-fMRI data may be capable of identifying patients with BSP, and regions from the SMN, cerebellum, and DMN may provide key insights into the underlying pathophysiology of BSP.

## Introduction

Idiopathic blepharospasm (BSP) is one of the most common adult-onset focal dystonia that manifests with indirect or persistent excessive involuntary orbicularis oculi contraction and blinking (1, 2). It has been reported that BSP can affect about 16–133 cases per million with a greater predisposition in females (3). Patients with BSP also have several non-motor symptoms, such as sensory symptoms (such as burning sensation and grittiness in the eyes) (4), psychiatric disorders (such as depression, anxiety, and obsessive-compulsive disorders) (5, 6), sleep disorders, and cognitive disturbances. Based on clear motor manifestations of patients, a diagnostic algorithm has been developed with 93% sensitivity and 90% specificity to distinguish BSP from other conditions of involuntary lid closure (7, 8). Several treatment options can be administered by clinicians to effectively manage the symptoms of BSP, but usually only with short-term efficacy (9). Therefore, the pathophysiological mechanism of BSP needs to be better understood to develop new treatments.

With the development of neuroimaging technology, functional and microstructural changes of the whole brain can now be visualized, which further improves the understanding of the neurophysiology of BSP. Resting-state functional MRI (rs-fMRI), which measures spontaneous neural activities based on the fluctuation of blood-oxygen-level-dependent (BOLD) signals (10), is an effective tool to explore the functional brain reorganization. The regional homogeneity (ReHo) has been used to assess local features of spontaneous brain activities, and significantly decreased ReHo was found in the insula, calcarine cortex, and superior medial frontal gyrus, while increased ReHo was found in the supplementary motor area (SMA) in patients with BSP relative to healthy controls (HCs) (11). The resting-state voxel-wise functional connectivity (FC) can reflect functional relationships between spatially separated cortical signals. Significantly decreased FC was shown in the superior medial prefrontal cortex (mPFC)/anterior cingulate cortex (ACC), while increased FC was shown in the postcentral gyrus/precentral gyrus/paracentral lobule/SMA and right superior frontal gyrus (SFG) that involved in the default mode network (DMN) and sensorimotor network (SMN) in patients with BSP compared with HCs (12). In our previous study, using the amplitude of low-frequency fluctuations (ALFF) to explore local cerebral activities, we found significantly decreased ALFF in the thalamus and increased ALFF in the orbitofrontal areas extending from the middle frontal gyrus (MFG) to the inferior frontal gyrus (IFG) in patients with BSP compared to HCs, suggesting abnormal sensorimotor integration and dysfunction of the thalamus in BSP (13). These results proposed that BSP is associated with functional alterations across different brain regions.

At present, more attention has been paid to changes in general brain networks, rather than specific brain regions. Graph theoretical analysis can be applied to examine the large-scale topology of brain networks. By definition, there are three kinds of networks: regular, random, and complex networks with their characteristics (14). The human brain network can be regarded as a complex network with high global and high local connectivity. The random network that is significantly less clustered (lower local connectivity) than the small-world network has approximately the same global connectivity as the small-world network (15). We explored the functional connectome in Parkinson's disease (PD) patients with and without mild cognitive impairment and have found disrupted topological organization in PD (16). This study explores the functional connectome in patients with BSP through rs-fMRI and graph theoretical analysis.

## Materials and Methods

### Participants

The West China Hospital of Sichuan University Clinical Trials and Biomedical Ethics Committee approved the study, and all participants gave written informed consent in the study. All patients were diagnosed based on the diagnostic criteria of BSP (7). Patients were excluded if they had (1) contraindication for MRI scans; (2) a history of other neurologic and psychiatric diseases; and (3) organic brain defect on T1- or T2-weighted images. Finally, 20 right-handed patients were recruited. In contrast, 20 right-handed age- and gender-matched HCs were recruited from the local community with no history of neurologic and psychiatric diseases and no organic brain defect on T1- or T2-weighted images. The demographic and clinical data, such as age, gender, and disease duration, were collected using a standard questionnaire by a movement disorder specialist during face-to-face interviews. Both severity and frequency of the involuntary orbicularis oculi muscle spasm were accessed according to the Jankovic rating scale (JRS) (1, 17). The JRS total score (0–8) is the sum of two parts: the JRS-severity score which ranges from 0 (=absence of severity) to 4 (=maximum severity) and the JRS-frequency score which ranges from 0 (=no frequency) to 4 (=maximum frequency). Only two patients with BSP were treated with botulinum neurotoxin (BoNT) 1 and 2 years ago, while the remaining patients with BSP have never received BoNT treatment. All patients with BSP have been using drugs, such as anticholinergics, benzodiazepines, and baclofen, to improve symptoms and withdrew medications for ~24 h before MRI scanning.

### MRI Acquisition and Preprocessing

The MRI was performed on a 3T scanner (Excite; GE, Milwaukee, WI, USA) with an eight-channel phased-array head coil. Anatomical images were acquired using a spoiled gradient-recalled sequence. The rs-fMRI (gradient-echo echo-planar imaging (EPI) sequence: repetition time/echo time (TR/TE) = 2,000/30 ms, flip angle (FA) = 90°, field of view (FOV) = 240 × 240 mm^2^, matrix size = 64 × 64, slice thickness = 5.0 mm) was performed. Supplementary Material reported the complete MRI protocol. One experienced neuroradiologist evaluated image abnormalities and verified image quality. Rs-fMRI data were processed with the Statistical Parametric Mapping (SPM12, http://www.fil.ion.ucl.ac.uk/spm) and Data Processing and Analysis for Brain Imaging (DPABI, http://rfmri.org/DPABI), and the preprocessing steps included removing the first 10 time points, slice timing corrections, spatial realignment, spatial normalization into the standard Montreal Neurological Institute (MNI) space and resample in 3 × 3 × 3 mm^3^ voxels, detrending, nuisance signal regression (such as the Friston 24 parameters, white matter signal, cerebrospinal fluid signal, and global signal), and band-pass filtering (0.01–0.08 Hz). All participants had less than 1.0 mm maximum displacement in the x-, y-, and z-planes and less than 1.0° of angular rotation about each axis. The data were also reprocessed without the global signal regression (GSR).

### Network Construction and Analysis

The complex network analysis, a new multidisciplinary approach, can characterize brain networks through a series of measures with neurobiological significance and computation simplicity (14). The network model consists of nodes and edges between any two nodes. A total of 160 regions of interest (ROIs) (as nodes) were defined by Dosenbach et al. (18) (see the Supplementary Table 1). Pearson correlations of the meantime series between any two ROIs (nodes) were defined as edges. The FC matrix (160 × 160) was computed for each subject. Through the toolbox of GRETNA v2.0 (http://www.nitrc.org/projects/gretna/), we could translate each FC matrix into a binary matrix according to the predefined threshold (10% ≤ sparsity ≤ 34% with the interval of 0.01) (19), and the area under the curve (AUC) over sparsity ranges was calculated independent of a single selected threshold (20). The following measures of network topology were calculated: (1) small-world parameters, clustering coefficient (*C*_p_) that can quantify the local interconnectivity of a network and the shortest path length (*L*_p_) that can quantify the integration of a network. By definition, γ ($=C_{p}^{real}/C_{p}^{rand}$) is the normalized *C*_p_; λ ($=L_{p}^{real}/L_{p}^{rand}$) is the normalized *L*_p_; and σ = γ / λ. The $C_{p}^{real}$ and $L_{p}^{real}\;$are, respectively, *C*_p_ and *L*_p_ derived from our “real” brain networks, and the $C_{p}^{rand}$ and $L_{P}^{rand}\;$represent the corresponding indices of 100 matched random networks. A small-world network should fulfill the following criteria: γ > 1 and λ ≈ 1 or σ > 1 (15); (2) Network efficiency parameters, global efficiency (*E*_glob_) that can indicate the efficiency of information transference across a network and local efficiency (*E*_loc_) that can indicate the capability of information exchange for each subgraph when the index node is eliminated; (3) the nodal parameter, nodal efficiency (*E*_nod_) that can evaluate the node importance for information communication in a network (see the Supplementary Material for details).

### Statistical Analysis and Classification Model

Student's *t*-test or the Chi-square test was used to detect differences of demographic data between groups, as appropriate. Global and nodal metrics were compared between patients with BSP and HCs using Wilcoxon rank-sum test. Multiple comparisons with FDR correction were performed in the nodal centrality.

The least absolute shrinkage and selection operator (LASSO) regression is an approach for variable selection in fitting a high-dimensional generalized linear model. To obtain classified variables and prevent overfitting results, LASSO regression model analysis was currently adopted to determine the most critical imaging features from the nodal centrality (*E*_nod_). Ten-fold cross-validation (CV) was used to determine the strength λ of the penalty term in LASSO. The minimum of the CV error was considered to obtain an optimal value of λ. The number of critical imaging features with the optimal model was determined by λ. A model for classifying BSP using the critical imaging features was constructed based on the corresponding regression coefficients obtained from LASSO regression. The receiver operating characteristic (ROC) curve was used to explore the sensitivity and specificity of the classification model. Accordingly, we referred to the Youden's index and defined an optimal value of predictive risk score. To validate the validity of the results, the model was applied in the testing sets. The testing sets were randomly sampled from the whole cohort by 19 times. The sample size was increased from 21 to 39. The AUC of ROC was calculated to compare the accuracy of the model in classifying BSP from HC. The implementation of the LASSO method was conducted through the “glmnet” package. All statistical analyses were performed in R version 3.6.3.

## Results

**Figure 1:**
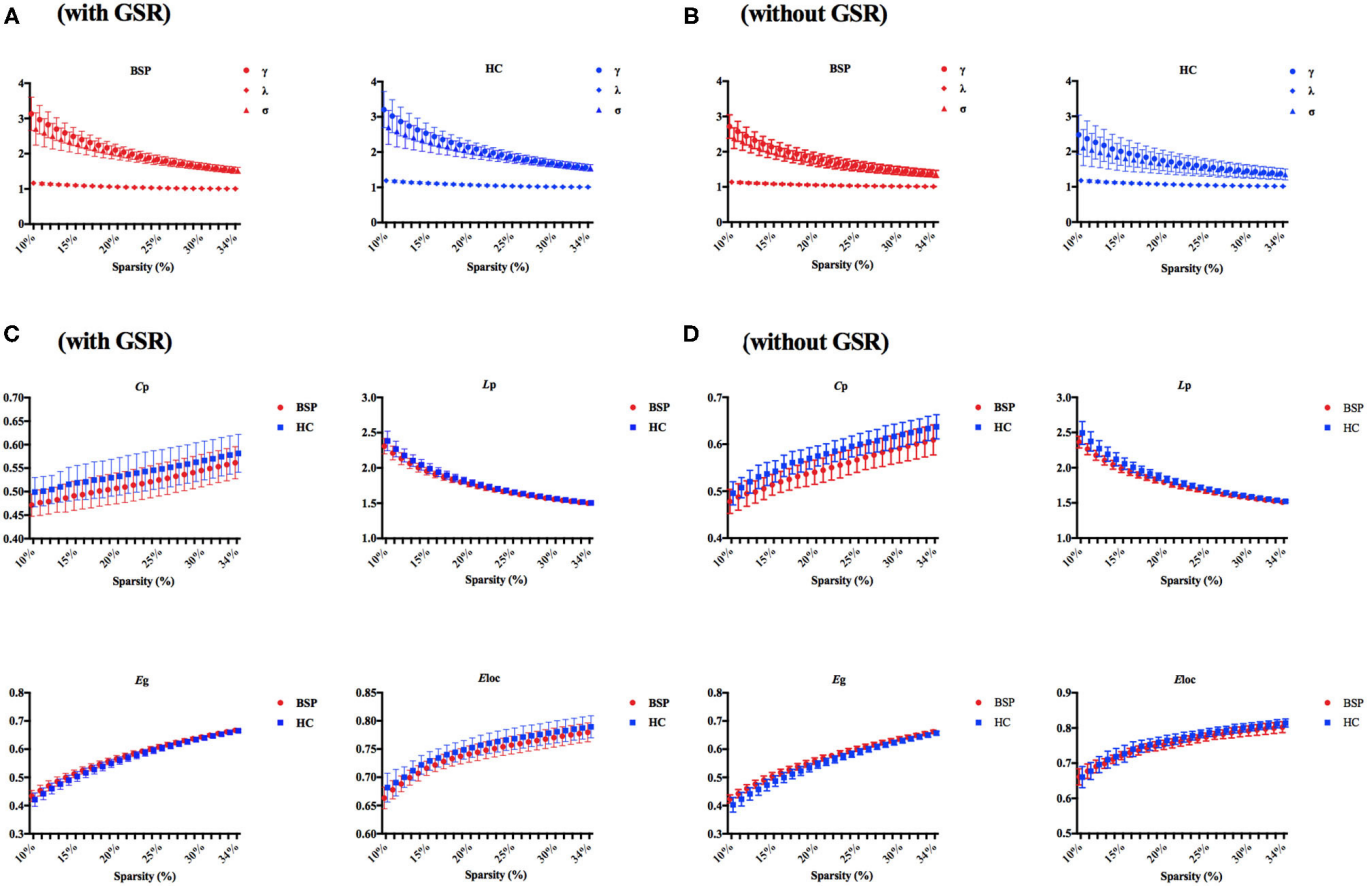
**(A)** Small-world properties in patients with BSP and HCs (with GSR); **(B)** small-world properties in patients with BSP and HCs (without GSR); **(C)** global topographic properties in BSP and HC groups with over the selected range of the sparsity threshold (with GSR); **(D)** global topographic properties in BSP and HC groups with over the selected range of the sparsity threshold (without GSR). BSP, blepharospasm; *C*_p_, clustering coefficient; *E*_glob_, global efficiency; *E*_loc_, local efficiency; GSR, global signal regression; HC, healthy control; *L*_p_, characteristic path length.

The demographic and clinical characteristics of participants are shown in Table 1. There was no significant difference in age and gender between patients with BSP and HCs. The typical small-world properties [γ > 1 and λ ≈ 1 or σ > 1; Figure 1A (with GSR); Figure 1B (without GSR)] and global parameters [*C*_p_, *L*_p_, *E*_glob_, and *E*_loc_; Figure 1C (with GSR); Figure 1D (without GSR)] were shown in patients with BSP and HCs over the selected range of the sparsity threshold.

**Table 1:** Demographic and clinical characteristics of the total samples.

**Parameter**	**Controls**	**BSP**	***P***
Number, *n*	20	20	–
Handedness of writing (Right: Left)	20: 0	20: 0	–
Age, years	53.00 ± 8.78	53.90 ± 8.64	0.746
Gender, Male/Female	5/15	4/16	0.705
Duration of disease, years	–	3.26 ± 2.40	–
JRS-severity sub-score	–	3.05 ± 0.83	–
JRS-frequency sub-score	–	2.80 ± 0.83	–
JRS-total score	–	5.85 ± 1.63	–

*BSP, blepharospasm; JRS, Jankovic rating scale*.

**Figure 2:**
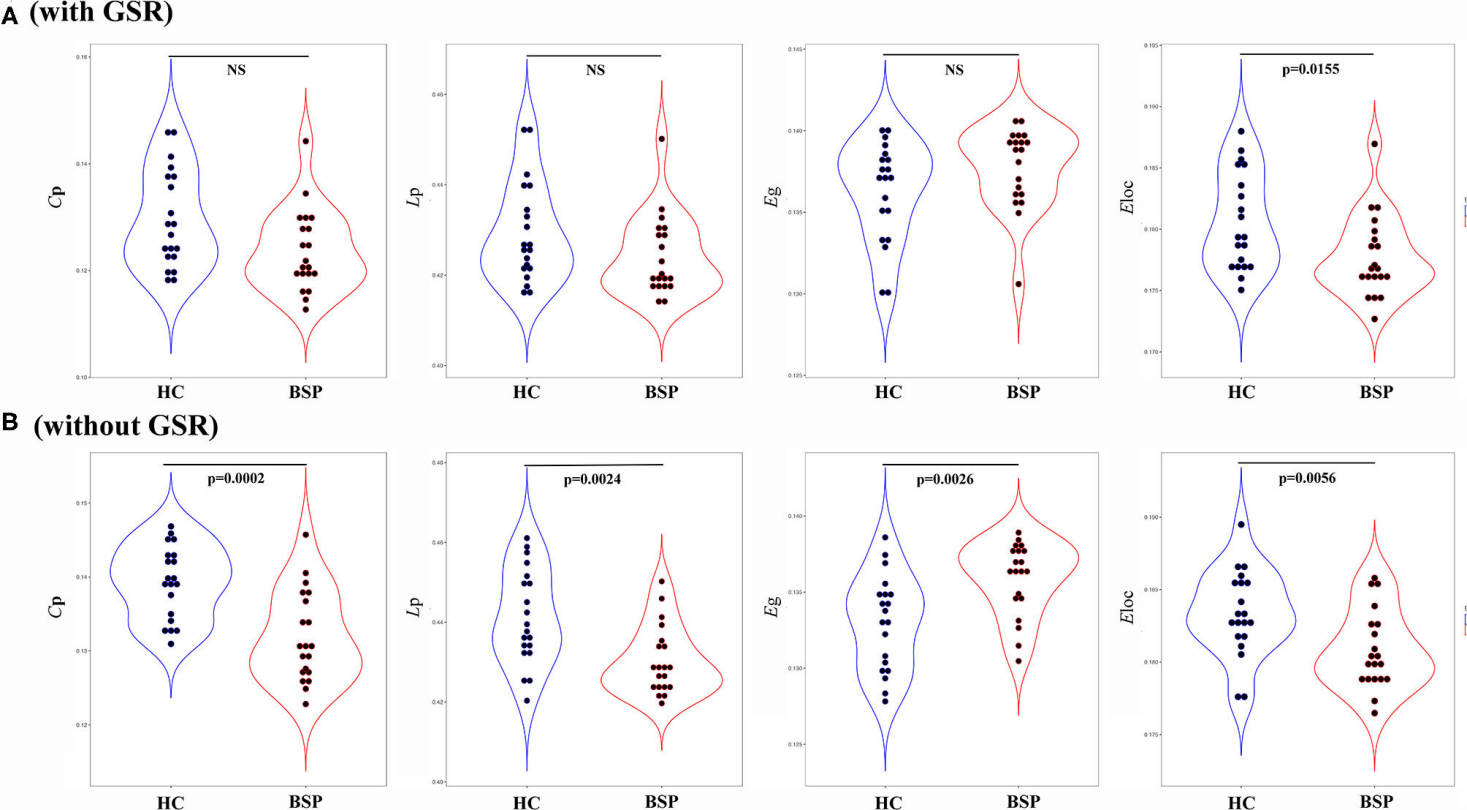
**(A)** Inter-group differences in global topographic properties (with GSR); **(B)** inter-group differences in global topographic properties (without GSR). Values on the *y*-axis represent the AUC of the graph indices across the range of the sparsity threshold. AUC, area under the curve; BSP, blepharospasm; *C*_p_, clustering coefficient; *E*_glob_, global efficiency; *E*_loc_, local efficiency; HC, healthy control; *L*_p_, characteristic path length.

For inter-group differences in global parameters with GSR, a decreased local connectivity was indicated by a reduced trend of clustering coefficient and significantly reduced local efficiency in patients with BSP relative to HCs (*C*_p_: *P*_BSP_ vs. _HCs_ = 0.052; *E*_loc_: *P*_BSP_ vs. _HCs_ = 0.015). Meantime, a decreased trend of path length and an increased trend of global efficiency were found in patients with BSP [*L*_p_: *P*_BSP_ vs. _HCs_ = 0.096; *E*_glob_: *P*_BSP_ vs. _HCs_ = 0.096; Figure 2A (with GSR)]. For inter-group differences in global parameters without GSR, a decreased local connectivity was presented by significantly reduced clustering coefficient and local efficiency in patients with BSP (*C*_p_: *P*_BSP_ vs. _HCs_ < 0.001; *E*_loc_: *P*_BSP_ vs. _HCs_ = 0.005), while increased global connectivity was shown by significantly reduced path length and significantly increased global efficiency in patients with BSP [*L*_p_: *P*_BSP_ vs. _HCs_ = 0.002; *E*_glob_: *P*_BSP_ vs. _HCs_ = 0.003; Figure 2B (without GSR)].

**Figure 3:**
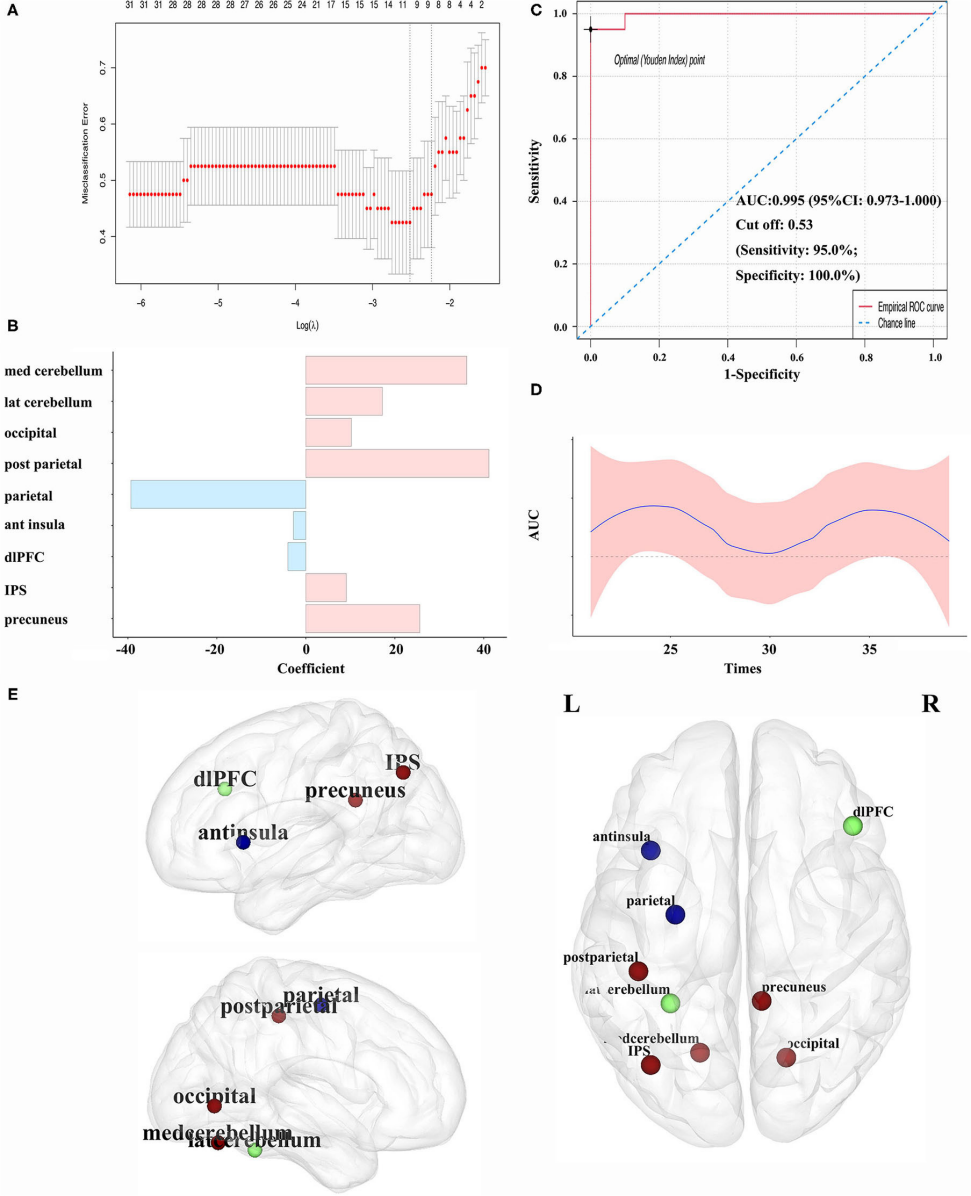
**(A)** Variables selection by LASSO regression in the classification model; **(B)** coefficient of each variable in the model; **(C)** the ROC curves in classifying patients with BSP for the model; **(D)** internal validation for the model. The testing sets were randomly sampled from the whole cohort by 19 times. The sample size was increased from 21 to 39; **(E)** abnormal nodal centralities in patients with BSP compared with HCs (the blue node representing the decreased *E*_nod_ in patients with BSP, the red node representing the increased *E*_nod_ in patients with BSP, and the green node representing no difference between patients with BSP and HCs). ant, anterior; AUC, area under the curve; BSP, blepharospasm; dlPFC, dorsolateral prefrontal cortex; *E*_nod_, nodal efficiency; HC, healthy control; IPS, intraparietal sulcus; LASSO, least absolute shrinkage and selection operator; lat, lateral; med, medial; post, posterior; ROC, receiver operating characteristic; ROI, region of interest.

Imaging features of nodal centrality (*E*_nod_) were entered into the LASSO method, and the classification model was constructed (Figure 3A). Imaging features from nine nodes were incorporated into the model, and four nodes within the SMN, cerebellum, and DMN held the prominent information. The coefficients are displayed in Figure 3B; Table 2. The AUCs were 0.995 (95% CI: 0.973–1.000). The sensitivity and specificity were 95 and 100%, respectively. The cutoff value was 0.5307 (Figure 3C). The randomly sampled internal data set has further proved the capability of the classification model. As we can see in Figure 3D, all the AUCs are fluctuated around the AUC of the model.

**Table 2:** Comparison of critical imaging features between patients with BSP and HCs and the corresponding coefficients.

**No**.	**Region**	**Hemishpere**	**Network**	**Nodal Efficiency**				**Coefficients^#^**	**Intercept^#^**
				**BSP**	**HC**	***P*** **value**	***q*** **value**		
**BSP > HC**									−12.582335
21	Precuneus	R	Default	0.1371 ± 0.0010	0.1284 ± 0.0088	0.0132*	0.0341	25.6232737	–
30	IPS	L	Default	0.1382 ± 0.0087	0.1317 ± 0.0073	0.0181*	0.0341	9.10143256	–
117	Post parietal/postcentral	L	Sensorimotor	0.1433 ± 0.0068	0.1354 ± 0.0110	0.0227*	0.0341	41.1562545	–
126	Occipital	R	Occipital	0.1397 ± 0.0063	0.1352 ± 0.0073	0.0195*	0.0341	10.2355905	–
143	Lat cerebellum	L	Cerebellum	0.1349 ± 0.0083	0.1302 ± 0.0070	0.0810	0.0911	17.2030057	–
150	Med cerebellum	L	Cerebellum	0.1441 ± 0.0090	0.1366 ± 0.0086	0.0095*	0.0341	36.1654607	–
**BSP < HC**	–	–	–	–	–	–	–	–	
42	dlPFC	R	Fronto-parietal	0.1349 ± 0.0081	0.1386 ± 0.0100	0.2315	0.2315	**–**4.0251895	–
62	Ant insula	L	Cingulo-opercular	0.1440 ± 0.0010	0.1502 ± 0.0069	0.0375*	0.0482	**–**2.7689898	–
98	Parietal/precentral	L	Sensorimotor	0.1389 ± 0.0094	0.1455 ± 0.0048	0.0039*	0.0341	**–**39.31437	–

* *Region considered abnormal*.

# *Equation with dependent variables and the corresponding coefficients in the regression model*.

*ant, anterior; BSP, blepharospasm; dlPFC, dorsolateral prefrontal cortex; HC, healthy control; IPS, intraparietal sulcus; L, left; lat, lateral; med, medial; post, posterior; R, right*.

Significant nodal features from the classification model were compared between patients with BSP and HCs, and significant changes were found in seven regions (Table 2; Figure 3E). Notably, for the above four core regions, patients with BSP showed significantly increased *E*_nod_ in the postcentral region within the SMN, decreased *E*_nod_ in the precentral region within the SMN, increased *E*_nod_ in the medial cerebellum, and increased *E*_nod_ in the precuneus within the DMN (Table 2).

## Discussion

In this study, characteristics of brain networks were identified in patients with BSP through the rs-fMRI and graph network analysis. It is generally known that GSR is a controversial pre-processing strategy for the rs-fMRI data. GSR can effectively remove global artifacts caused by motion and respiration, but it can also get rid of some distributed neural signals and introduce negative correlation biases (21). In our study, the pre-processing strategies with or without GSR did not substantially change the differences in the network level between patients with BSP and HCs. On this global level, the decreased *C*_p_ and *E*_loc_ indicated deficits in specialized processing within densely interconnected clusters of the brain regions or networks and suggested the reduced functional segregation of the whole brain in the BSP group (14). Furthermore, the small-world network reflects an optimal balance of functional integration and segregation (high global and high local connectivity), but the random network displays high global and low local connectivity. Taken together, compared with HCs, brain networks in patients with BSP may be shifting toward a more random network organization, which may describe a disease state of the whole brain in patients with BSP and is similar to other movement disorders, such as PD (22).

Applying nodal features from extensive brain regions, this study proposed the classification model with a high discrimination power. A previous rs-fMRI study showed that global-brain functional connectivity (GFC) values *in priori* brain regions within the SMN could discriminate patients with BSP from HCs with optimal accuracy (12). In the current study, nodal features from the whole brain were selected, nodal efficiency values of nine significant regions resulting from mathematical consequence were survived, and then a classification model with high specificity and high sensitivity was proposed. Significantly, nodes from the SMN, cerebellum, and DMN held the critical information in descending order to discriminate patients with BSP from healthy participants. Our findings suggested that the pathophysiological mechanism of BSP was closely related to the re-organization of large-scale functional brain networks, and regions from the SMN, cerebellum, and DMN may provide key insights into the underlying pathophysiology of BSP.

Regional alterations in the SMN can partly explain abnormal movements in patients with BSP (12, 23). The performance of movements requires preparation, execution, and monitoring mechanisms. The first two are coded by the motor system and the latter by the sensory system, which can properly integrate motor and sensory information into a feedback loop (24). The regional deficits in the SMN in movement disorders can cause abnormalities in this sensory-motor loop with a negative impact on the sensory-motor integration mechanisms and accurate movements. The decreased nodal activities of the precentral region and increased nodal activities of the postcentral region observed in the current study may reflect the abnormal peripheral inhibition, show the more complex information processing, and result in excessive muscle contraction and unnecessary behavior in patients (25).

Another feature was abnormal nodal activities in the cerebellum, mainly in the form of elevation. The cerebellum exhibited hypermetabolism for patients with BSP during wakefulness in a previous PET study (26). The current opinion is that dystonic movements can be driven by neuronal dysfunction originating from the cerebellum (27). The significant increase in nodal activities of the cerebellum could be interpreted as a compensatory change (2), and the well-known regional abnormality in the cerebellum has been regarded as a common base for the propagation of larger-scale network abnormalities that contributed to the development of dystonic characteristics (28).

The DMN, a novel and recently appreciated brain system, such as posterior cingulate gyrus (PCC) and precuneus, can express high levels of activities in the resting state and participate in internal modes of cognition (29). Previous neuropsychological studies found that patients with focal dystonia had a broad range of cognitive deficits, such as executive and memory dysfunction (30–32), which might be related to the disrupted functional activities in the DMN.

Accumulating neuroimaging evidence indicates BSP as a network disorder originating from dysfunction of multiple brain regions (as nodes) within different brain networks and abnormal communications between different nodes (33, 34), which explains the heterogeneity of motor and non-motor symptoms in patients with BSP. Future studies need to focus on the interaction mechanism between the different brain regions, the kinds of motor and non-motor manifestations, and further shed light on the genetic basis.

Some limitations of this study should be noted. First, the generalization of results in this study was limited by the relatively small sample size. In addition, BSP patients with a history of other neurologic and psychiatric diseases were excluded in the current study; hence, these results did not apply to the general population of patients with BSP. Second, the assessment of non-motor manifestations of patients with BSP was absent in this study, which should be further considered using comprehensive rating tools. Third, our patients were all medicated and withdrawn from their medication for about 24 h before MRI scanning, which might affect the brain function and be counted as a limitation. It is important to note that the possible role of treatment in this process has not been fully clarified. At last, only cross-sectional changes of the rs-fMRI parameters were investigated without performing longitudinal assessments.

## Conclusion

We found that the network model of patients with BSP may gradually change from the small-world network to the random network with reduced functional segregation. Moreover, the disrupted nodal centralities derived from rs-fMRI data may be capable of identifying patients with BSP. The nodal efficiency from four regions within the SMN, cerebellum, and DMN held the critical information. Our results may provide insights into the underlying pathophysiology of BSP.

## Author's Note

Idiopathic blepharospasm (BSP) is a common adult-onset focal dystonia. It has been reported that BSP can affect about 16–133 cases per million with a greater predisposition in females. Several treatment options can be administered by clinicians to effectively manage the symptoms of BSP, but usually only with short-term efficacy. Therefore, the pathophysiological mechanism of BSP needs to be better understood to develop new treatments. At present, more attention has been paid to changes in general brain networks, rather than specific brain regions. Under the resting-state functional MRI (rs-fMRI) and graph theoretical analysis, we found that the network model of patients with BSP may gradually change from the small-world network to the random network. Moreover, the nodal efficiency may be capable of identifying patients with BSP. Our results may provide insights into the underlying pathophysiology of BSP.

## Data Availability Statement

The datasets presented in this article are not readily available because the data that support the findings of this study are available from the corresponding author upon reasonable request. Requests to access the datasets should be directed to hfshang2002@126.com.

## Ethics Statement

The studies involving human participants were reviewed and approved by the West China Hospital of Sichuan University Clinical Trials and Biomedical Ethics Committee. The patients/participants provided their written informed consent to participate in this study.

## Author Contributions

All authors listed have made a substantial, direct, and intellectual contribution to the work and approved it for publication.

## Funding

This present study was supported by the 1.3.5 project for disciplines of excellence, West China Hospital, Sichuan University (ZYJC18038 to HS).

## Conflict of Interest

The authors declare that the research was conducted in the absence of any commercial or financial relationships that could be construed as a potential conflict of interest.

## Publisher's Note

All claims expressed in this article are solely those of the authors and do not necessarily represent those of their affiliated organizations, or those of the publisher, the editors and the reviewers. Any product that may be evaluated in this article, or claim that may be made by its manufacturer, is not guaranteed or endorsed by the publisher.
