# Supplementary material for: Impaired Topographic Organization in Patients With Idiopathic Blepharospasm
Source: Front Neurol. 2022 Jan 12;12:708634. doi: 10.3389/fneur.2021.708634 (PMC8791229; doi:10.3389/fneur.2021.708634)
Supplement: Supplementary file 1 [file Data_Sheet_1.docx]

**MRI acquisition**

The MR imaging was performed on a 3T scanner (Excite; GE, Milwaukee, WI) with an eight-channel phased-array head coil. High-resolution T1-weighted images were acquired via a volumetric three-dimensional spoiled gradient recall sequence (Repetition time/echo time (TR/TE) = 8.5/3.4 ms, flip angle (FA) = 12°, field of view (FOV) = 240 × 240 mm^2^, slice thickness = 1.0 mm). The final 156 contiguous axial slices with a slice thickness of 1.0 mm were produced by using the FOV (240 × 240 mm^2^) and an acquisition matrix comprising 256 readings of 128 phase encoding steps. The final matrix size was automatically interpolated in-plane to 512×512 with an in-plane resolution of 0.47×0.47 mm^2^. The resting-state functional MR images were obtained via a gradient-echo echo-planar imaging sequence (EPI) (TR/TE = 2000/30 ms, FA = 90°, FOV = 240 × 240 mm^2^, matrix size = 64×64, slice thickness = 5.0 mm [no slice gap], voxel size = 3.75 × 3.75 × 5 mm^3^). The functional run contained 200 image volumes with 30 axial slices in each brain volume. The scanning was performed in darkness, and all participants were instructed to be relaxed, keep their eyes closed, and do not fall asleep (confirmed immediately after the examination) during the MRI acquisition. Earplugs and cushions were used to minimize the scanner noise and head movement, respectively.

**Network construction and analysis**

1. Small-world and global topographic parameters

These measures included clustering coefficient (*C*_p_), characteristic path length (*L*_p_), normalized clustering coefficient (γ), normalized characteristic path length (λ), small-worldness (σ), global efficiency (*E*_glob_) and local efficiency (*E*_loc_).

For a given graph *G* with *N* nodes, the *C*_p_ is defined as ([Watts and Strogatz, 1998](#_ENREF_5)) (*D*_nod_(i) is the degree of node i, *E*_i_ is the number of edges in *G*i):

$$C_{p}=\frac{1}{N}\sum_{I\in G} \frac{E_{i}}{D_{\mathrm{nod}}(i)(D_{\mathrm{nod}}\left( i \right)-1)/2}$$

The *L*_p_ is defined as ([Newman, 2003](#_ENREF_3)) (*L_ij_* is the shortest path length between nodes *i* and *j*):

$$L_{P}=\frac{1}{\frac{1}{N(N-1)}\left( \sum_{j\neq i\in G} \frac{1}{L_{\mathrm{ij}}} \right)}$$

The *E*_glob_ is defined as ([Latora and Marchiori, 2001](#_ENREF_2)):

$$E_{\mathrm{glob}}=\frac{1}{N(N-1)}\sum_{j\neq i\in G} \frac{1}{L_{\mathrm{ij}}}$$

The *E*_loc_ is defined as ([Latora and Marchiori, 2001](#_ENREF_2)):

$$E_{\mathrm{loc}}(i)={E_{\mathrm{glob}}\left( Gi \right) ；E}_{\mathrm{loc}}=\frac{1}{N}\sum_{i\in G} E_{\mathrm{loc}}(i)$$

Taken together, *C*_p_ and *E*_loc_ measure the local cliquishness of a network, and *L*_p_ and *E*_glob_ measure the overall routing efficiency of a network.

1. Nodal topographic parameters

The nodal efficiency (*E*_nod_) is defined as ([Achard and Bullmore, 2007](#_ENREF_1)):

$$E_{\mathrm{nod}}=\frac{1}{N-1}\sum_{j\neq i\in G} \frac{1}{L_{\mathrm{ij}}}$$

**Reference:**

Achard, S., and Bullmore, E.T. (2007). Efficiency and cost of economical brain functional networks. *Plos Computational Biology* 3(2)**,** 174-183.

Latora, V., and Marchiori, M. (2001). Efficient behavior of small-world networks. *Phys Rev Lett* 87(19)**,** 198701.

Newman, M.E. (2003). Mixing patterns in networks. *Phys Rev E Stat Nonlin Soft Matter Phys* 67(2 Pt 2)**,** 026126.

Rubinov, M., and Sporns, O. (2010). Complex network measures of brain connectivity: Uses and interpretations. *Neuroimage* 52(3)**,** 1059-1069.

Watts, D.J., and Strogatz, S.H. (1998). Collective dynamics of 'small-world' networks. *Nature* 393(6684)**,** 440-442.

Zhang, J., Wang, J., Wu, Q., Kuang, W., Huang, X., He, Y., et al. (2011). Disrupted Brain Connectivity Networks in Drug-Naive, First-Episode Major Depressive Disorder. *Biological Psychiatry* 70(4)**,** 334-342.
