# Supplementary material for: Impaired Topographic Organization in Patients With Idiopathic Blepharospasm
Source: Front Neurol. 2022 Jan 12;12:708634. doi: 10.3389/fneur.2021.708634 (PMC8791229; doi:10.3389/fneur.2021.708634)
Supplement: Supplementary file 2 [file Table_1.docx]

Table S1. The labels, regions, corresponding subnetworks and coordinates of the regions of interest (ROIs).

|  |  |  |  | MNI-coordinates | | |
| --- | --- | --- | --- | --- | --- | --- |
| Labels | Regions | Hemishpere | Subnetwork | x(mm) | y(mm) | z(mm) |
| 1 | vmPFC | R | default | 6 | 64 | 3 |
| 2 | mPFC | / | default | 0 | 51 | 32 |
| 3 | aPFC | L | default | -25 | 51 | 27 |
| 4 | vmPFC | R | default | 9 | 51 | 16 |
| 5 | vmPFC | L | default | -6 | 50 | -1 |
| 6 | vmPFC | L | default | -11 | 45 | 17 |
| 7 | vmPFC | R | default | 8 | 42 | -5 |
| 8 | ACC | R | default | 9 | 39 | 20 |
| 9 | vlPFC | R | default | 46 | 39 | -15 |
| 10 | sup frontal | R | default | 23 | 33 | 47 |
| 11 | sup frontal | L | default | -16 | 29 | 54 |
| 12 | inf temporal | R | default | 52 | -15 | -13 |
| 13 | inf temporal | L | default | -59 | -25 | -15 |
| 14 | post cingulate | R | default | 1 | -26 | 31 |
| 15 | fusiform | R | default | 28 | -37 | -15 |
| 16 | precuneus | L | default | -3 | -38 | 45 |
| 17 | post cingulate | L | default | -8 | -41 | 3 |
| 18 | inf temporal | L | default | -61 | -41 | -2 |
| 19 | occipital | L | default | -28 | -42 | -11 |
| 20 | post cingulate | L | default | -5 | -43 | 25 |
| 21 | precuneus | R | default | 9 | -43 | 25 |
| 22 | precuneus | R | default | 5 | -50 | 33 |
| 23 | post cingulate | L | default | -5 | -52 | 17 |
| 24 | post cingulate | R | default | 10 | -55 | 17 |
| 25 | precuneus | L | default | -6 | -56 | 29 |
| 26 | post cingulate | L | default | -11 | -58 | 17 |
| 27 | angular gyrus | R | default | 51 | -59 | 34 |
| 28 | angular gyrus | L | default | -48 | -63 | 35 |
| 29 | precuneus | R | default | 11 | -68 | 42 |
| 30 | IPS | L | default | -36 | -69 | 40 |
| 31 | occipital | L | default | -9 | -72 | 41 |
| 32 | occipital | R | default | 45 | -72 | 29 |
| 33 | occipital | L | default | -2 | -75 | 32 |
| 34 | occipital | L | default | -42 | -76 | 26 |
| 35 | aPFC | R | fronto-parietal | 29 | 57 | 18 |
| 36 | aPFC | L | fronto-parietal | -29 | 57 | 10 |
| 37 | vent aPFC | R | fronto-parietal | 42 | 48 | -3 |
| 38 | vent aPFC | L | fronto-parietal | -43 | 47 | 2 |
| 39 | vlPFC | R | fronto-parietal | 39 | 42 | 16 |
| 40 | dlPFC | R | fronto-parietal | 40 | 36 | 29 |
| 41 | ACC | L | fronto-parietal | -1 | 28 | 40 |
| 42 | dlPFC | R | fronto-parietal | 46 | 28 | 31 |
| 43 | vPFC | L | fronto-parietal | -52 | 28 | 17 |
| 44 | dlPFC | L | fronto-parietal | -44 | 27 | 33 |
| 45 | dFC | R | fronto-parietal | 40 | 17 | 40 |
| 46 | dFC | R | fronto-parietal | 44 | 8 | 34 |
| 47 | dFC | L | fronto-parietal | -42 | 7 | 36 |
| 48 | IPL | L | fronto-parietal | -41 | -40 | 42 |
| 49 | IPL | R | fronto-parietal | 54 | -44 | 43 |
| 50 | post parietal | L | fronto-parietal | -35 | -46 | 48 |
| 51 | IPL | L | fronto-parietal | -48 | -47 | 49 |
| 52 | IPL | L | fronto-parietal | -53 | -50 | 39 |
| 53 | IPL | R | fronto-parietal | 44 | -52 | 47 |
| 54 | IPS | L | fronto-parietal | -32 | -58 | 46 |
| 55 | IPS | R | fronto-parietal | 32 | -59 | 41 |
| 56 | aPFC | R | cingulo-opercular | 27 | 49 | 26 |
| 57 | vPFC | R | cingulo-opercular | 34 | 32 | 7 |
| 58 | ACC | L | cingulo-opercular | -2 | 30 | 27 |
| 59 | vFC | R | cingulo-opercular | 51 | 23 | 8 |
| 60 | ant insula | R | cingulo-opercular | 38 | 21 | -1 |
| 61 | dACC | R | cingulo-opercular | 9 | 20 | 34 |
| 62 | ant insula | L | cingulo-opercular | -36 | 18 | 2 |
| 63 | basal ganglia | L | cingulo-opercular | -6 | 17 | 34 |
| 64 | mFC | / | cingulo-opercular | 0 | 15 | 45 |
| 65 | vFC | L | cingulo-opercular | -46 | 10 | 14 |
| 66 | basal ganglia | L | cingulo-opercular | -20 | 6 | 7 |
| 67 | basal ganglia | R | cingulo-opercular | 14 | 6 | 7 |
| 68 | vFC | L | cingulo-opercular | -48 | 6 | 1 |
| 69 | mid insula | R | cingulo-opercular | 37 | -2 | -3 |
| 70 | thalamus | L | cingulo-opercular | -12 | -3 | 13 |
| 71 | thalamus | L | cingulo-opercular | -12 | -12 | 6 |
| 72 | thalamus | R | cingulo-opercular | 11 | -12 | 6 |
| 73 | mid insula | R | cingulo-opercular | 32 | -12 | 2 |
| 74 | mid insula | L | cingulo-opercular | -30 | -14 | 1 |
| 75 | basal ganglia | R | cingulo-opercular | 11 | -24 | 2 |
| 76 | post insula | L | cingulo-opercular | -30 | -28 | 9 |
| 77 | temporal | R | cingulo-opercular | 51 | -30 | 5 |
| 78 | post cingulate | L | cingulo-opercular | -4 | -31 | -4 |
| 79 | fusiform | R | cingulo-opercular | 54 | -31 | -18 |
| 80 | precuneus | R | cingulo-opercular | 8 | -40 | 50 |
| 81 | parietal | R | cingulo-opercular | 58 | -41 | 20 |
| 82 | temporal | R | cingulo-opercular | 43 | -43 | 8 |
| 83 | parietal | L | cingulo-opercular | -55 | -44 | 30 |
| 84 | sup temporal | R | cingulo-opercular | 42 | -46 | 21 |
| 85 | angular gyrus | L | cingulo-opercular | -41 | -47 | 29 |
| 86 | temporal | L | cingulo-opercular | -59 | -47 | 11 |
| 87 | TPJ | L | cingulo-opercular | -52 | -63 | 15 |
| 88 | frontal | R | sensorimotor | 58 | 11 | 14 |
| 89 | dFC | R | sensorimotor | 60 | 8 | 34 |
| 90 | vFC | L | sensorimotor | -55 | 7 | 23 |
| 91 | pre-SMA | R | sensorimotor | 10 | 5 | 51 |
| 92 | vFC | R | sensorimotor | 43 | 1 | 12 |
| 93 | SMA | / | sensorimotor | 0 | -1 | 52 |
| 94 | frontal | R | sensorimotor | 53 | -3 | 32 |
| 95 | precentral gyrus | R | sensorimotor | 58 | -3 | 17 |
| 96 | mid insula | L | sensorimotor | -42 | -3 | 11 |
| 97 | precentral gyrus | L | sensorimotor | -44 | -6 | 49 |
| 98 | parietal | L | sensorimotor | -26 | -8 | 54 |
| 99 | precentral gyrus | R | sensorimotor | 46 | -8 | 24 |
| 100 | precentral gyrus | L | sensorimotor | -54 | -9 | 23 |
| 101 | precentral gyrus | R | sensorimotor | 44 | -11 | 38 |
| 102 | parietal | L | sensorimotor | -47 | -12 | 36 |
| 103 | mid insula | R | sensorimotor | 33 | -12 | 16 |
| 104 | mid insula | L | sensorimotor | -36 | -12 | 15 |
| 105 | temporal | R | sensorimotor | 59 | -13 | 8 |
| 106 | parietal | L | sensorimotor | -38 | -15 | 59 |
| 107 | parietal | L | sensorimotor | -47 | -18 | 50 |
| 108 | parietal | R | sensorimotor | 46 | -20 | 45 |
| 109 | parietal | L | sensorimotor | -55 | -22 | 38 |
| 110 | precentral gyrus | L | sensorimotor | -54 | -22 | 22 |
| 111 | temporal | L | sensorimotor | -54 | -22 | 9 |
| 112 | parietal | R | sensorimotor | 41 | -23 | 55 |
| 113 | post insula | R | sensorimotor | 42 | -24 | 17 |
| 114 | parietal | R | sensorimotor | 18 | -27 | 62 |
| 115 | parietal | L | sensorimotor | -38 | -27 | 60 |
| 116 | parietal | L | sensorimotor | -24 | -30 | 64 |
| 117 | post parietal | L | sensorimotor | -41 | -31 | 48 |
| 118 | temporal | L | sensorimotor | -41 | -37 | 16 |
| 119 | temporal | L | sensorimotor | -53 | -37 | 13 |
| 120 | sup parietal | R | sensorimotor | 34 | -39 | 65 |
| 121 | occipital | L | occipital | -18 | -50 | 1 |
| 122 | occipital | L | occipital | -34 | -60 | -5 |
| 123 | occipital | R | occipital | 36 | -60 | -8 |
| 124 | temporal | R | occipital | 46 | -62 | 5 |
| 125 | occipital | L | occipital | -44 | -63 | -7 |
| 126 | occipital | R | occipital | 19 | -66 | -1 |
| 127 | occipital | R | occipital | 17 | -68 | 20 |
| 128 | occipital | R | occipital | 39 | -71 | 13 |
| 129 | occipital | R | occipital | 29 | -73 | 29 |
| 130 | occipital | L | occipital | -29 | -75 | 28 |
| 131 | occipital | L | occipital | -16 | -76 | 33 |
| 132 | occipital | R | occipital | 9 | -76 | 14 |
| 133 | occipital | R | occipital | 15 | -77 | 32 |
| 134 | occipital | R | occipital | 20 | -78 | -2 |
| 135 | post occipital | L | occipital | -5 | -80 | 9 |
| 136 | post occipital | R | occipital | 29 | -81 | 14 |
| 137 | post occipital | R | occipital | 33 | -81 | -2 |
| 138 | post occipital | L | occipital | -37 | -83 | -2 |
| 139 | post occipital | L | occipital | -29 | -88 | 8 |
| 140 | post occipital | R | occipital | 13 | -91 | 2 |
| 141 | post occipital | R | occipital | 27 | -91 | 2 |
| 142 | post occipital | L | occipital | -4 | -94 | 12 |
| 143 | lat cerebellum | L | cerebellum | -28 | -44 | -25 |
| 144 | lat cerebellum | L | cerebellum | -24 | -54 | -21 |
| 145 | inf cerebellum | L | cerebellum | -37 | -54 | -37 |
| 146 | lat cerebellum | L | cerebellum | -34 | -57 | -24 |
| 147 | med cerebellum | L | cerebellum | -6 | -60 | -15 |
| 148 | inf cerebellum | L | cerebellum | -25 | -60 | -34 |
| 149 | inf cerebellum | R | cerebellum | 32 | -61 | -31 |
| 150 | med cerebellum | L | cerebellum | -16 | -64 | -21 |
| 151 | lat cerebellum | R | cerebellum | 21 | -64 | -22 |
| 152 | med cerebellum | R | cerebellum | 1 | -66 | -24 |
| 153 | inf cerebellum | L | cerebellum | -34 | -67 | -29 |
| 154 | med cerebellum | L | cerebellum | -11 | -72 | -14 |
| 155 | inf cerebellum | R | cerebellum | 33 | -73 | -30 |
| 156 | med cerebellum | R | cerebellum | 5 | -75 | -11 |
| 157 | med cerebellum | R | cerebellum | 14 | -75 | -21 |
| 158 | inf cerebellum | L | cerebellum | -21 | -79 | -33 |
| 159 | inf cerebellum | L | cerebellum | -6 | -79 | -33 |
| 160 | inf cerebellum | R | cerebellum | 18 | -81 | -33 |

Key words: ant, antieror; aPFC, anterior prefrontal cortex; dACC, dorsal anterior cingulate cortex; dFC, dorsal frontal cortex; dlPFC, dorsolateral prefrontal cortex; inf, inferior; IPL, inferior parietal lobule; IPS, intraparietal sulcus; lat, lateral; med, medial; mid, middle; post, posterior; SMA, supplementary motor area; sup, superior; TPJ, temporo-parietal junction; vent, ventral; vFC, ventral frontal cortex; vlPFC, ventrolateral prefrontal cortex; vmPFC, ventromedial prefrontal cortex; vPFC, ventral prefrontal cortex.
